# Hypomyopathic Dermatomyositis Presenting with Idiopathic CD4 T-lymphocytopenia and Delayed Anti-MDA5 Positivity

**DOI:** 10.7759/cureus.4133

**Published:** 2019-02-25

**Authors:** Maria C Mijares, Adam S Aldahan, Hector H Gonzalez, Nabil Benhayoun, David Alboukrek

**Affiliations:** 1 Internal Medicine, Florida Atlantic University Charles E. Schmidt College of Medicine, Boca Raton, USA; 2 Dermatology, University of Minnesota, Minneapolis, USA; 3 Rheumatology, Florida Atlantic University Charles E. Schmidt College of Medicine, Boca Raton, USA

**Keywords:** hypomyopathic dermatomyositis, anti-mda5, mda5, cd4 t-lymphocytopenia, interstitial lung disease

## Abstract

A 47-year-old Haitian male with no known past medical history was admitted to the hospital for gradually progressive dyspnea, nonproductive cough, and weight loss. He also endorsed a one-year history of joint pains. He was febrile and tachycardic and in mild respiratory distress. Other pertinent physical examination findings included diffuse inspiratory crackles, digital ulcers, and symmetric swelling of the wrists, elbows, shoulders, and knees. He was found to have a right basilar consolidation on chest computed tomography (CT) and was placed on antibiotics for presumptive pneumonia. His CD4 count was 158 cells per microliter despite testing negative for human immunodeficiency virus (HIV). A thorough infectious workup was unrevealing, and he did not improve with antibiotics. He had a weakly positive anti-nuclear antibody (ANA) with an otherwise negative rheumatologic workup. Creatinine kinase and aspartate aminotransferase were mildly elevated in the absence of overt muscle weakness. A myositis panel, including melanoma differentiation-associated protein five (anti-MDA5) antibody, was negative at the time. He was discharged on a short course of prednisone without a definitive diagnosis. He returned several months later with worsening respiratory symptoms. At this time, a lung biopsy revealed interstitial lung disease. Repeat myositis panel demonstrated anti-MDA5 positivity. The patient was also found to have new-onset non-ischemic heart failure with reduced ejection fraction. A diagnosis of hypomyopathic dermatomyositis was made based on clinical, laboratory, and imaging findings. The patient was restarted on prednisone, and mycophenolate mofetil was subsequently initiated for maintenance therapy.

## Introduction

Hypomyopathic dermatomyositis is an autoimmune syndrome characterized by specific cutaneous findings, non-erosive bilateral polyarthritis, and subclinical elevations of muscle enzymes. The presence of antibodies to melanocyte differentiation-associated gene five (MDA5) is associated with the development of rapidly progressing interstitial lung disease [1-2]. Herein, we describe a case of hypomyopathic dermatomyositis presenting with delayed anti-MDA5 positivity as well as idiopathic cluster of differentiation four (CD4) T-lymphocytopenia.

## Case presentation

A 47-year-old Haitian male with no known past medical history presented to the emergency department in May 2018 with a one-year history of gradually progressive dry cough and dyspnea. He endorsed associated fevers, night sweats, anorexia, and symmetric polyarthralgias in the hands, wrist, elbows, shoulders, and knees. Review of systems was negative for weight loss, alopecia, dry eyes, dry mouth, mouth sores, and photosensitivity. The patient denied sick contacts, recent travel, tick bites, pets, or exposure to birds. He works as a cook, and denied any industrial exposure, alcohol consumption, smoking, or illicit drug use. He has no known family history. He has no allergies and takes no medications or supplements.

**Figure 1:**
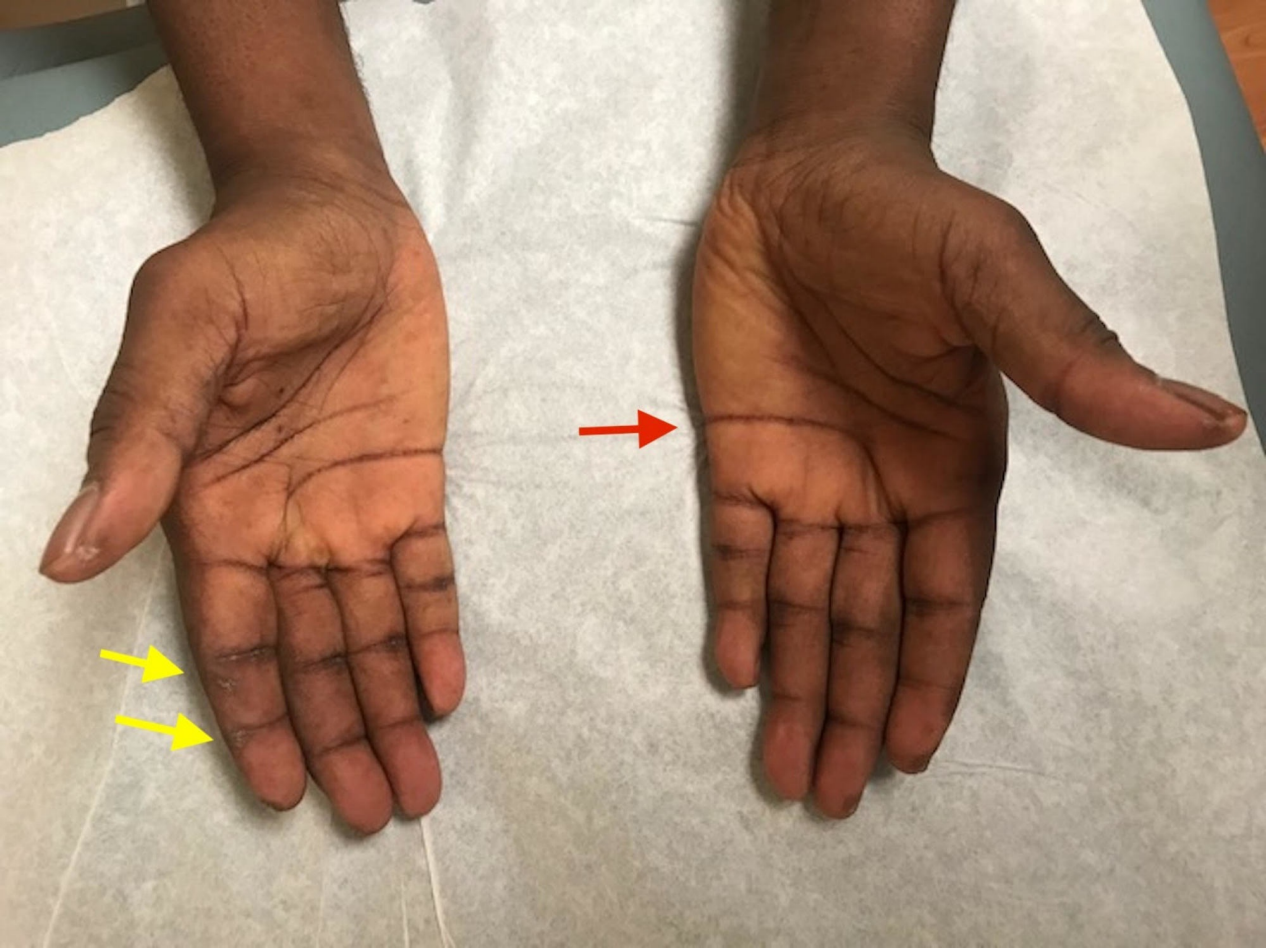
Fissures and hyperpigmentation of palmar creases (red arrow); marked ulceration of the right second fingertip (yellow arrows)

On initial presentation, he was in mild respiratory distress, tachycardic, and febrile. He was normotensive and saturating 98% on room air. Pulmonary exam revealed fine inspiratory crackles diffusely over the bilateral lung fields. He did not have any abnormal heart sounds or murmurs. The abdomen was soft and non-tender without organomegaly. Musculoskeletal exam revealed symmetric swelling and tenderness of the bilateral wrists, elbows, shoulders, and knees. Several metacarpophalangeal (MCP) and proximal interphalangeal (PIP) joints of the hands were also affected. There was no muscle tenderness or decreased strength or sensation. Several shallow ulcers and fissures were present on the fingertips along with hyperpigmentation of the knuckles and creases of palms (Figure 1).

Electrocardiogram was normal aside from sinus tachycardia. Initial laboratory studies demonstrated a marked lymphopenia, erythrocyte sedimentation rate 40 and aspartate aminotransferase 95. Otherwise, renal, liver, and thyroid tests were normal. Creatinine kinase was near the upper limit of normal at 179 units per liter. Chest computed tomography (CT) revealed a large right basilar consolidation, diffuse ground-glass opacities, small bilateral effusions, and diffusely and enlarged mediastinal lymph nodes. No honeycombing or cavitary lesions were identified. The patient was started empirically on antibiotics for pneumonia. Blood, sputum, and urine cultures were negative. His CD4 returned at 158 cells per microliter, although human immunodeficiency virus (HIV) screening was negative despite repeated testing of both antibodies and polymerase chain reaction (PCR). Subsequent infectious workup including mycoplasma, legionella, tuberculosis, hepatitis, syphilis, and parvovirus, was negative. Initial rheumatologic workup revealed a weakly positive anti-nuclear antibody (ANA) titer of 1:80 dilution. Anti-rheumatic factor (RF), anti-cyclic citrullinated protein (CCP), anti-neutrophilic cytoplasmic autoantibodies (ANCA), anti-smith, anti-ribonucleoprotein (RNP), and myositis panel were nonreactive. Fiberoptic bronchoscopy with bronchoalveolar lavage was performed for diagnostic clarity, which revealed no fluid, hemorrhage, or tumor. Lavage samples were negative for infections including pneumocystis. Transthoracic echocardiogram was unremarkable without evidence of heart failure, valvular abnormalities, or endocarditis.

During hospitalization, the patient continued to have dyspnea and fevers as well as worsening joint pains. The differential diagnosis at this time included inflammatory arthritis, seronegative rheumatoid arthritis, idiopathic inflammatory myopathies, anti-synthetase syndrome, and cryptogenic organizing pneumonia. Less likely etiologies included allergic interstitial pneumonia, sarcoidosis, vasculitis, paraneoplastic syndrome, lymphoma, cytomegalovirus (CMV) pneumonia, and human T-lymphotropic virus infection. Due to the progressive arthralgias, lack of response to antibiotics, and unremarkable infectious workup, an autoinflammatory disease was favored. Rheumatology was consulted and prednisone was initiated with a subsequent rapid symptomatic improvement of his polyarthralgia in the subsequent days, although his dyspnea on exertion only minimally improved. The patient was discharged home on a prednisone taper but returned to the hospital several months later with worsening dyspnea and recurrent polyarthritis.

After a repeated bronchoscopy was unrevealing, a thoracoscopic wedge biopsy was performed and was consistent with cryptogenic organizing pneumonia. Repeat myositis panel at this time showed positive antibodies to MDA5 by line immunoassay. Autoimmune testing was otherwise unchanged. Peripheral blood smear and bone marrow biopsy showed no evidence of blood cell dyscrasias. Imaging of the head, chest, abdomen, and pelvis was unremarkable. A repeat echocardiogram showed new-onset heart failure with an ejection fraction of 30% to 35%. Cardiac ischemic workup was negative. Cardiac magnetic resonance imaging (MRI) was negative for wall motion abnormalities or infiltrating diseases.

A diagnosis of dermatomyositis (DM) was made based on clinical findings, anti-MDA5 positivity, and lung biopsy. The subclinical muscle enzyme elevation led to a more specific diagnosis of hypomyopathic DM. Despite the absence of muscle weakness on examination, the slight elevations of muscle enzymes such as creatinine kinase and aspartate aminotransferase prevent this from being classified as amyopathic DM. This diagnosis was obscured by the initial negative myositis panel as well as the idiopathic CD4 T-lymphocytopenia, which is not well described in hypomyopathic DM patients. The patient was placed on immunosuppression therapy with mycophenolate mofetil and continued on low dose prednisone. He continued close outpatient follow-up with rheumatology, pulmonology, and cardiology.

## Discussion

Dermatomyositis (DM) is a type of idiopathic inflammatory myopathy that presents with hallmark cutaneous findings and myositis. Skin manifestations of DM include heliotrope rash, Gottron papules, mechanics hands, non-inflammatory skin ulcers, palmar papules, and oral ulcers. The myositis usually presents clinically as muscle weakness and is associated with various muscle specific antibodies. Adult DM has a peak onset in the fifth decade with a 2:1 female to male ratio [1]. The subgroups of DM include classic DM, post-myopathic DM, DM sine dermatitis, and clinical amyopathic DM. Classic DM presents with characteristic skin findings and muscle weakness associated with elevated muscle enzymes. Post-myopathic DM occurs in classic DM patients with persistent skin findings despite resolution in myositis. Conversely, DM sine dermatitis has muscle biopsy findings consistent with DM in the absence of skin findings. Clinically amyopathic DM is defined as classic skin manifestations of DM with little to no muscle symptoms in the first six months of disease onset [2]. Clinically amyopathic DM encompasses 20% of all DM patients and can be further broken down into amyopathic DM and hypomyopathic DM. Amyopathic DM has no muscle weakness and no signs of muscle inflammation on serology, electromyography, or muscle biopsy.

Hypomyopathic DM, on the other hand, does have signs of muscle inflammation in the absence of clinical muscle weakness. Clinically amyopathic DM may develop overt muscle weakness within 10 years after skin disease onset. For this reason, it is recommended to repeat muscle enzyme testing intermittently to evaluate disease progression [3]. The diagnosis of clinically amyopathic DM cannot be made in patients with skin findings who are immediately started high dose systemic immunosuppressive therapy, which may mask any underlying myositis. Additionally, certain drugs such as hydroxyurea, non-steroidal anti-inflammatory drugs, antibiotics, and statins may produce DM-specific cutaneous findings. These patients are not classified as clinically amyopathic DM [4]. There are several types of well-described myositis-specific antibodies associated with DM. The most common include anti-transfer ribonucleic acid (tRNA) synthetases such as anti-Jo-1. Since these autoantibodies have cytoplasmic and not nuclear targets, a negative or low titer ANA does not preclude the diagnosis of DM. Myositis-associated antibodies such as anti-Ro, anti-Scl, and anti-U1 RNP can be seen in overlap syndromes between DM and other connective tissue diseases [4].

MDA5 has been more recently identified as a myositis-specific antigen in clinically amyopathic DM patients. It is highly associated with the development of interstitial lung disease (ILD) [5]. The anti-MDA5 antibody is postulated to develop via molecular mimicry, although the exact mechanism is not entirely understood [6]. This antibody portends a poor prognosis, and the ILD seen in these cases is often refractory to steroid and immunosuppressant therapy. There may be additional genetic factors that play a role in the severity of ILD, as Asian patients with anti-MDA5 antibodies seem to have the highest rates of rapidly progressing ILD [6]. Nevertheless, the association has also been described in Caucasian patients [7]. Given the emerging evidence in the literature, patients with DM may benefit from routine testing for the anti-MDA5 antibody. In cases of anti-MDA5 positivity, treatment with immunosuppressant agents should be started immediately to halt the progression of the disease.

The patient, in this case, was diagnosed with hypomyopathic DM based on a combination of clinical, laboratory, imaging findings. Although the patient did not have many of the classical cutaneous findings of DM, digital ulcers have been described in clinically amyopathic DM. Additionally, there may be an association between skin ulcers and rapidly progressive ILD in these patients [8]. The hyperpigmentation seen on his knuckles and palmar creases may have represented post-inflammatory changes. Cutaneous features are usually recognized clinically, but a skin biopsy may be performed in questionable cases. Histopathology of cutaneous DM includes vacuolar interface dermatitis with mucin deposition in the dermis, although this may also be seen in cutaneous lupus erythematosus [9]. The patient also had characteristic non-erosive symmetric polyarthritis, including swelling of several MCP and PIP joints, wrist, elbows, shoulders, and knees. Despite this, he did not have muscle weakness either reported in history or elicited on examination. The presence of cutaneous findings in the absence of overt muscle weakness classified this patient as clinically amyopathic DM. Given his mild elevation of creatinine kinase and aspartate aminotransferase, hypomyopathic DM was the most appropriate designation [4]. Confirmatory testing such as electromyography or muscle biopsy was deemed unnecessary in this patient without clinical muscle weakness.

The most likely differential diagnosis, in this case, was anti-synthetase syndrome, which is a rare autoimmune syndrome characterized by fever, non-erosive polyarthralgia, ILD, myopathies, cutaneous manifestations, and Raynaud’s phenomenon. The most common antibody encountered is anti-Jo-1, a type of anti-tRNA synthetase antibody, positive in about 75% of cases. ILD is found in at least 70% of patients and is considered one of the most important prognostic factors [10]. This syndrome has recently been recognized as its own entity on the spectrum of idiopathic inflammatory myopathies. Despite the similar clinical presentation to DM, the diagnostic criteria for anti-synthetase syndrome require an anti-synthetase antibody to be present. The patient, in this case, had negative anti-synthetase antibodies and positive anti-MDA5 antibodies, which ultimately clinched the diagnosis of DM.

 Cardiac involvement is rare in patients with DM, occurring in 3% to 6% of cases [1]. The most common cardiac manifestation is non-ischemic heart failure. As with ILD, cardiac involvement represents a poor prognostic factor and should be monitored closely [11]. Proposed mechanisms of heart disease include cardiac myositis by circulating inflammatory cells, as well as concomitant viral myocarditis. Less likely mechanisms include targeted destruction of cardiac cells by circulating autoantibodies. Cardiac MRI is often helpful in uncovering viral or infiltrative causes; however, in this patient, cardiac MRI was unrevealing. The idiopathic CD4 T-lymphocytopenia seen in this case remains unexplained. Low CD4 in the serum is often associated with HIV infection, although several other infectious agents have also been implicated. Though rarely reported with DM, lymphocytopenia may be an independent risk factor for rapidly progressive ILD in clinically amyopathic DM patients, even if the anti-MDA5 antibodies are negative [8]. CD4 T-lymphocytopenia is also rarely associated with systemic inflammation or internal malignancy. One study of 47 patients with DM reported a median CD4 count of 383 cells per microliter. The authors reported that 12 patients developed pneumocystis pneumonia after steroid treatment and suggested that prophylactic antibiotics may be warranted in DM patients with low CD4 counts [12].

DM patients have the highest rates of malignancies of all the idiopathic inflammatory myopathies. Therefore, it is important to screen for internal malignancies in newly diagnosed patients [13]. Anti-MDA5 antibodies are not thought to be independently associated with internal malignancy [14]. The patient, in this case, had initial imaging of the head, chest, abdomen, and pelvis without evidence for malignancy. He was referred for routine colonoscopy.

## Conclusions

This case of cryptogenic organizing pneumonia presenting in conjunction with new onset heart failure, idiopathic CD4 T-lymphocytopenia, and delayed anti-MDA5 positivity represents a rare constellation of symptoms in a newly diagnosed hypomyopathic dermatomyositis patient. Hypomyopathic dermatomyositis is a rare syndrome that is difficult to diagnose. This case describes several rare manifestations of hypomyopathic DM including CD4 T-lymphocytopenia. The initially negative myositis panel highlights the discordance between serology and disease severity. DM patients can have significant disease progression even in the absence of detectable circulating antibodies. It is important to repeat myositis panels and maintain a low threshold to treat if clinical suspicion is high. Routine testing for anti-MDA5 antibodies is warranted in DM patients given the association with rapidly progressive ILD. Further studies are needed to better understand the relationship between autoantibodies and disease progression in these patients. Therefore, clinicians should have a low threshold to treat, and negative antibodies should not preclude early initiation of treatment if clinical suspicion is high.
